# Inflammatory markers and indicators of systemic endotoxemia in patients with treatment-resistant schizophrenia

**DOI:** 10.1192/j.eurpsy.2023.568

**Published:** 2023-07-19

**Authors:** S. A. Zozulya, I. N. Otman, I. A. Anikhovskaya, D. V. Tikhonov, V. G. Kaleda, M. Y. Yakovlev, T. P. Klyushnik

**Affiliations:** ^1^Laboratory of Neuroimmunology, Mental Health Research Centre; ^2^Laboratory of Systemic Endotoxinemia and Shock, Research Institute of General Pathology and Pathophisiology; ^3^Department of Youth Psychiatry, Mental Health Research Centre, Moscow, Russian Federation

## Abstract

**Introduction:**

Elevated levels of lipopolysaccharide (LPS) in circulation support chronic inflammation, which is involved in the pathological process in the brain and may be a contributing factor to treatment resistance in schizophrenia.

**Objectives:**

To compare inflammatory markers and indicators of systemic endotoxemia (SE) in patients with treatment-resistant schizophrenia and in those with a good response to treatment.

**Methods:**

The study involved 34 patients with schizophrenia (27±7,5 years) (F20) in an acute psychotic state: 15 patients with TRS (non-responders), 19 patients responded to treatment with reduced symptoms (responders). The markers of systemic inflammation (leukocyte elastase (LE) and a1-proteinase inhibitor (α1-PI) activity, CRP concentration, antibodies (Abs) to S100B and myelin basic protein) and the indicators of SE (LPS level and Abs to LPS) were determined in the blood of patients.

**Results:**

The responders showed a significant increase in LE and α1-P1 activity (p<0.001), CRP concentration (p<0.05), and Abs to neuroantigens (p<0.05) compared to controls. LPS levels did not differ from control values. In non-responders, a moderate increase in LE and α1-PI activities (p<0.05) and a significant increase in CRP concentration (p=0.01) were accompanied by no significant differences in Abs to neuroantigens. These patients had elevated LPS level and Abs to LPS deficiency compared with both responders (p<0.01) and controls (p<0.05).

**Conclusions:**

The identified spectra of systemic inflammation markers, elevated LPS level, and insufficient anti-endotoxin immunity in patients with treatment-resistant schizophrenia may be related to endotoxin tolerance. Further research in this field can help develop new approaches to overcoming resistance to therapy in patients with schizophrenia.

**Disclosure of Interest:**

None Declared

